# Thirty-Seven Cases of Urinary Calculus

**Published:** 1874-10

**Authors:** W. F. Westmoreland

**Affiliations:** Professor of Theory and Practice of Surgery in Atlanta Medical College; Atlanta, Ga.


### THIRTY-SEVEN CASES OF URINARY CALCULUS

TREATED BY W. F. WESTMORELAND, M.D.,
*Professor of Theory and Practice of Surgery in Atlanta Medical College.*

*Read before the Georgia Medical Association, April, 1874.*

The object of this paper is a synopsis of thirty-seven cases of urinary calculus, in thirty-three of which the stone was removed from the bladder by lateral lithotomy; one from the bladder by the hopogastric or high operation; one from the urethra by external urethotomy; one from the urethra by internal section of a stricture, with Westmoreland's urethrotome; and one from the urethra by Sir Henry Thompson's divulsor.

Of the thirty-seven cases, there were thirty-six males, and one female. Five were between sixty and seventy years of age, one was thirty-five, and the remaining thirty-one were under twenty years of age; the youngest was only eighteen months old when the operation was performed. Thirty-four recovered, and three died. Of the three fatal cases, one was sixty-eight, one was sixty-one, and the other ten years of age.

I first propose to present the prominent features of the fatal cases, with cause of death, etc.; and of the cases that recovered to discuss only those that presented peculiarities, accidents occurring during the treatment, or other points that would likely interest the profession.

Mr. S., of Carroll county, Georgia, aged sixty-eight years, had suffered for a number of years with calculus. When I saw him first, in 1869, he was greatly debilitated from long and continual suffering. Upon introducing the sound a large stone was readily detected. The bladder and urethra were extremely irritable; the walls of the bladder greatly thickened; the prostate gland enlarged. There was a pocket or *cul-de-sac* behind the enlarged middle lobe of the prostate, in which the stone rested. There was constant dribbling of urine, with frequent and violent efforts to expel the contents of the bladder. He had been confined to his bed for several months; daily exacerbation of fever; appetite capricious, often entirely wanting. I declined to operate, but suggested a course of treatment, with a view of allaying the intense irritation of the urethra and bladder, and building up his general health.

In three or four months I was again called to see Mr. S., and found, upon the whole, no improvement. The irritability of the urethra and bladder was to some extent relieved, but he was more feeble. He made the most touching appeal for me to relieve him by removing the stone. He insisted that the operation should be performed at once, contending that his sufferings were such that death was far preferable to such an existence. I attempted to dissuade him from the operation, telling him frankly that his chances for recovery after the operation were so slight that I did not feel that I would be entirely justifiable in its performance. In reply to my arguments to dissuade him from surgical interference, he said that I had admitted that there was no hope of relief from pain without the removal of the stone; that life at best could only be a few weeks or months of agony, and, as there was a possibility, however gloomy, of recovery, or even a partial exemption from his suffering, he felt that I should not abandon him to such a cruel fate. After a free conference with his family I decided to operate; thus yielding any pride I may have had of a brilliant series of lithotomies for statistical record to the appeals of suffering humanity.

Lithotripsy was made impracticable by the extreme irritability of the urethra and bladder, the enlargement of the prostate gland, and the deformity of the bladder above alluded to.

Assisted by Drs. Wilson, Slaughter, Hodgson, Connally and others, the patient was placed fully under the influence of chloroform, and the lateral section made. The stone was readily seized with the forceps, and removed without difficulty. As I was heartily congratulating myself upon the conclusion of the operation, imagine my surprise and consternation, in further exploring the bladder, to find another pocket, or *cul-de-sac*, in front and above the symphisis pubis, in which were found six calculi, ranging in size from a hazel nut to an inch in diameter.

Their removal was attended with considerable difficulty. To reach them with the forceps, it was necessary to make firm pressure above the pubes, thus displacing the deformed bladder toward the spine. They were soft or friable, the majority of them breaking down under the forceps, thus protracting the operation to an unusual length of time in the removal of the fragments and *debris* of the crushed calculi.

The patient came out from under the chloroform with but slight shock, and for twenty hours expressed himself as feeling decidedly more comfortable than for many years past. On the second day acute cystitis set in, and the patient died on the fourth day after the operation.

Mr. S., of Clay county, Alabama, sixty-one years of age, had suffered for four years with symptoms of stone in the bladder. In the past twelve or fifteen months his sufferings for several weeks at a time had been intense. He was considerably emaciated, but still able to be up most of the time; was only confined to his bed during the acute paroxysms. Exploration revealed a stone an inch and a half in diameter, resting in a pocket posterior to the enlarged middle lobe of the prostate gland.

Assisted by Dr. A. W. Griggs and others, the patient was chloroformed, and the lateral section made with a lithotome, and the stone readily seized, but was crushed under the forceps into innumerable fragments in attempting its removal. A considerable length of time was required for the removal of the fragments and *debris* from the bladder and wound. The shock was considerable, and the sufferings of the patient great immediately after the operation, requiring the free administration of anodynes to make him at all comfortable. The patient died the fourth day after the operation, of supposed cystitis.

My third and last death from lithotomy occurred in a boy ten years of age, living in the vicinity of Oxford, Georgia. I saw him with, and was assisted in the operation by, my friends, Dr. A. Means and Dr. Evans, of Oxford, Georgia. We found the little sufferer greatly exhausted, and in a dying condition, from long and continued suffering. A stone an inch in diameter was readily detected. The question that then presented itself was whether surgical interference should be attempted in his then exhausted condition. The attempt to improve his condition had already been tried without result. All thought it possible, and some even probable, that he would die on the table if an operation was attempted. On the other hand, it was evident that he could live but a few days or weeks at best, unless the stone was removed. What was to be done? Should we withhold surgical interference, and see the patient die in a few days from intense suffering, or should we operate, with many chances of the patient dying on the table? All the facts were laid before the family of the little sufferer. They favoring the operation, I again laid aside my pride of statistics, and removed the stone by the lateral method. My patient never recovered from the shock, and died the second day after the operation.

The above three cases are the only deaths in the thirty-seven in which I have operated. It will be seen that two were extreme cases—one in a dying condition when the operation was performed, with barely a hope of success. Both would have been rejected by the majority of surgeons, and certainly by those who pride themselves upon their brilliant statistical record. The other case was in much better condition, but was over sixty years of age.

Of the cases that recovered, the first accident that I met with was in master W., of Atlanta; eight years of age; good constitution, and in every particular a most favorable case for lithotomy. The lateral operation was performed, and a stone three-fourths of an inch in diameter removed without difficulty. Nothing unusual occurred at the time of the operation. Three days after the operation my little patient was violently attacked with dysentery, and for several days was dangerously ill. After the second day the inflammation extended to the small intestines, the discharges from the bowel being less dysenteric, assuming more the character of diarrhœa. Dysentery was prevailing in the city at the time. On the eighth day after the lithotomy, the mother sent for me in great haste, and on my arrival informed me that fecal matter had passed through the wound. This continued for a number of days. The inflammation of wound and bowel, with the exhausted and irritable condition of patient, was such that I did not examine the parts thoroughly for two or three weeks after the accident was first noticed. At that time I found an opening in the bowel, in the vicinity of the prostate gland and neck of bladder, at least an inch in diameter, and rather oblong in shape. The wound in the perineum had not yet closed, so the patient passed water by the urethra, perineal opening, and by the bowel. The patient had a tardy recovery; was confined to bed for two months or more. The wound in the perineum finally closed, and the patient recovered, but empties his bladder even now, nineteen years after the operation, partly by the urethra and partly by the bowel. He has positively refused any surgical interference with the view of closing the opening in the bowel. He enjoys good health, is stout and active, and no one would suspect in him such a trouble. Was the rectum wounded when the operation was performed? or was it a slough, the result of the acute dysentery? If the bowel had been opened at the time of operation, would we not have had a show before the eighth day? My impression at the time was that the rectum was slightly wounded, but not through the entire thickness of the bowel; that the large opening was a slough, the result of the dysentery.

In 1863 I was called to Troup county, Georgia, to operate on two brothers, sons of Mrs. T. One was sixteen and the other fourteen years of age. Assisted by Drs. Griggs and Ridley, I operated on both the same day and the same hour. The lateral method was adopted in both, and a stone of peculiar conformation was removed from each. The peculiarity of the calculi was the projections studding the surface. They resembled in conformation the sweet-gum burr, many of the projections on one of the stones being the fourth of an inch long. They were of the mulberry order. The projections were so extensive in the oldest boy that it became necessary to extend the incision with a bistoury to remove it from the bladder. Both recovered without unpleasant symptoms.

The next case that I will present is of interest, from the size of the stone and the mode of its removal. Master D., of Tennessee, twelve years of age, had suffered for a number of years from stone in the bladder; was greatly emaciated and exhausted from long-continued suffering; had been confined to his bed for a number of months. An exploration revealed the existence of a large stone; bladder and urethra intensely irritable; constant dribbling of urine. The case was regarded as very unpromising, but as surgical interference offered the only hope, I decided to operate.

Assisted by Dr. Benjamin Franklin, then of Philadelphia, Tennessee, the patient was chloroformed and the lateral incision made with the lithotome. The stone was found to fill the entire bladder, was the size of a goose egg, and about the shape. The bladder was firmly contracted upon it. The immense size of the stone was not suspected before the incision was made, as the exploration was rather imperfect, being made without anæsthetic, with an excessively irritable bladder, in a greatly exhausted patient.

It was evident that the stone could not be removed through any opening that could be made through the prostatic portion of the bladder without first breaking it down. I then introduced my lithotriptor, and, after grasping the stone in its smallest diameter, imagine my consternation to find that the calculus was so large that the screw of my instrument would not act on the blades. This was truly a dilemma. I had a lateral incision into the bladder, that would not permit extraction; a stone too large either to be crushed by my lithotriptor, or to be removed in the existing condition of the bladder, by the hypogastric operation, without extending the incision into its peritoneal covering. After a few moments' reflection, it occurred to me that I might possibly reduce the diameter of the stone by biting off pieces with the ordinary lithotomy forceps. The effort was made, and, to my great joy, I found that I could gradually reduce the diameter in this way. After forty minutes of constant nibbling, frequently changing the position of the stone, it was found sufficiently reduced to permit my lithotriptor to act upon it. It was now readily crushed, and the fragments removed with the forceps. The operation required an hour and ten minutes. The patient was kept fully under the influence of chloroform during the entire operation. The shock was not so great as we expected after such a trying ordeal. The patient recovered without an unfavorable symptom, and in two months was able to attend school.

Master P., of Atlanta, Georgia, six years of age, had suffered with gravel from early infancy, and from the commencement of his trouble there had been constant incontinence of urine, with occasional violent contractions of the bladder. Lateral lithotomy was performed before the class of the Atlanta Medical College, and a small stone removed. He rapidly recovered from the operation, with a relief to all previous trouble, except the incontinence, which continued as before the operation, under various plans of treatment, for two years, when, by a system of education, more than by the result of any medication, the bladder would retain the urine for an hour or two, and void it at will.

About this time I lost sight of the case, but some years later I learned that five years after the first operation another stone was removed from the bladder, followed by the same incontinence as after the first operation, but he had improved in this particular as he had grown older. Was the incontinence in this case from habit, the bladder having never been distended? or from defect, the result of operation? or was it the result of the presence of other calculi? I frequently examined him after the first operation, but found no irritation of urethra or bladder to account for the trouble, nor was I able to detect a stone.

The following is the only case of urinary calculus that I have ever met with in a female:

Miss J., seven years of age, had suffered for four years with the trouble, and for eight or ten months severely. Exploration established the presence of a stone of large size—so large that its removal by dilatation was not discussed. The child was poorly developed, and, after a careful examination of the vagina and urethra, the hypogastric or high operation was decided upon. This operation was performed during the war, when instrument makers were not accessible, so that our instruments in this case were, to a great extent, improvised.

Assisted by Drs. Brown, D'Alvigny, and others, the little patient was chloroformed, and the anterior wall of the bladder was laid bare by a vertical incision immediately above the pubes, cutting through the linea alba, fascia transversalis, and exposing the loose subfacial cellular tissues covering the anterior wall. In the absence of a *sonde-a-darde*, an ordinary male catheter of small size was introduced per urethram into the bladder, and the fundus forced up on the point of the catheter. An incision with a bistoury was now made an inch long; a hook, made by bending a metallic bougie, was then introduced through the incision, thus fixing the bladder; the catheter was removed, and, with a small pair of lithotomy forceps, the stone, an inch in diameter, readily removed. The wound was kept open, and a catheter introduced for the purpose of draining the bladder; but, from the extreme irritability of the urethra and great restlessness of the patient, it was found necessary to abandon it. At this time I belonged to the army medical corps, and, soon after the operation, was ordered to a distant State, and did not see my patient again until she was well. I was informed by Dr. Brown and Dr. D'Alvigny, who had charge of the patient after I left, that an abscess formed in the vicinity of the wound in the subfacial cellular tissue, producing a train of constitutional symptoms rather alarming. The abscess opened into the wound, and the symptoms disappeared.

Mr. S., of Lumpkin county, Georgia—aged seventy-one—powerful frame, and well preserved. Had been operated on, and a stone of considerable size removed, one or two years before. An exploration revealed the existence of another stone.

Assisted by Drs. Flewellen and Connally, the lateral section was made, and two calculi were removed—one oblong, and an inch and a half in the largest diameter; the other irregular in shape, and not so large. The patient was put to bed in good condition, the shock being slight.

Two hours later I was sent for in great haste, the messenger stating that the patient was bleeding freely. Upon my arrival I found that he had bled ten or twelve ounces, and was still bleeding freely. Upon examination I found a small artery at the upper portion of the incision, in the vicinity of the bulb. The point was touched freely with the muriated tincture of iron; a female catheter introduced into the bladder, and the wound packed with lint saturated with the same tincture. There was no further hemorrhage, and the old gentleman had a good recovery.

Mr. H., nineteen years of age; had suffered with gravel for four years; in good condition for operation. Lateral lithotomy was performed, and a medium-sized stone removed from the bladder. No accident during the operation, and but little hemorrhage. Four hours later an active hemorrhage set in, becoming retrocedent. I saw him half an hour after hemorrhage commenced, and found him suffering greatly, with frequent and violent efforts to expel the contents of the bladder. Small clots of blood were expelled at each effort. This was before I knew the styptic qualities of muriated tincture of iron. I resorted to the *canule-a-chemise*, repacking it two or three times without arresting the flow. I then introduced a female catheter, and, procuring the persulphate, packed the wound with lint saturated with a solution of this salt. The first packing greatly lessened the bleeding; the second completely and permanently arrested it, and the patient hád a good recovery.

Mr. L., of Forsyth county, Georgia, aged thirty-five; had suffered at intervals for a number of years with gravel. For the past year his suffering had been almost continuous. Exploration revealed the presence of a large stone.

Assisted by Drs. A. W. Griggs, Todd, and McMillen, of West Point, Georgia, the patient was chloroformed, and the lateral section made with the lithotome. The stone, which was very large, was readily grasped with the forceps, but, being very friable, composed of phosphate of lime, was crushed into innumerable fragments in attempting its removal. Several of the fragments were crushed in attempting their removal, leaving a mass of powdered stone in the bladder and wound after all the fragments of any size had been removed. A stream of tepid water, introduced by means of a catheter, thoroughly cleansed the bladder and wound of all foreign matter. The operation was greatly protracted by this accident, and was followed by an unusual reaction. A warm bath and the free administration of opiates promptly relieved the irritation, and the patient had a good recovery.

The following is the youngest subject upon which I have performed the operation of lithotomy, and well illustrates the principle that the younger the subject, every thing being equal, the more certain and rapid the recovery.

Master C., of Atlanta, aged eighteen months, had suffered for a year or more with symptoms of stone. The introduction of the sound at once revealed the existence of a stone of small size. The lateral section was made with a small lithotome, and a calculus the size of a hazel-nut removed. There was but a slight reaction following the operation, and in a week the patient was well.

In two cases, one twelve and the other sixteen years of age, where the calculi were large and the tissues unyielding, I produced fragmentation of the stone by means of the lithotriptor, introduced through the incision, and then removed the fragments with the lithotomy forceps. Both cases recovered promptly.

The above fifteen cases embrace all the interesting features or accidents occurring either before or after the operation in the thirty-four cases in which I have performed the operation of lithotomy. The remaining nineteen cases were patients ranging from six to eighteen years of age. Lateral lithotomy with the lithotome was made in each. No case presented any unusual features—all recovering promptly and without accident.

Of the three cases in which the calculi were found in the urethra, different methods in each case were adopted for their removal; and as such cases are often annoying, and consequently of interest to the surgeon, I will record them in the order in which they occurred.

Mr. ———, of Fulton county, Georgia, forty-eight years of age, had suffered for years with a supposed stricture of the urethra. Upon examination, I found a stone as large as a medium-sized pea firmly fixed in the bulbous portion of the urethra. It was evident, from the history of the trouble and the character of the tissues in the vicinity, that it had been in that position for a length of time—perhaps for years. An incision was made through the integuments and urethra, and the calculus removed. The urethra at that point had become sacculated, the stone being almost completely enveloped by the mucous membrane. The wound healed without trouble. A bougie was used for a number of months for the purpose of overcoming the stricture.

Mr. B., of Alabama, aged sixty years—greatly exhausted and emaciated by long and continual suffering—had suffered intensely for several weeks. Often there was incontinence of urine for several days at a time. Upon examination, found a perceptible enlargement of the urethra an inch and a half from the meatus urinarius, evidently caused by a calculus. I attempted to reach the point with a medium-sized sound, but the instrument was arrested by a stricture before reaching the foreign body. I then introduced my urethrotome, and readily passed the director through the stricture; the blade was now projected, and a section of the stricture made at three different points. The removal of the instrument was followed by a gush of urine and a dozen or more small calculi, with complete relief of all the sufferings of my patient. A No. 12 bougie was daily passed through the stricture, with instructions to introduce it once or twice a week for a number of months. Mr. B. left for his home in ten days, entirely relieved of all his troubles.

Judge C., of Atlanta, sixty years of age, had suffered for several years with urethral trouble. I had, three or four years before, performed perineal section for the relief of stricture and an extensive abscess, probably the result of the arrest of a gravel in the urethra. Judge C. had passed several small gravel after the perineal section, and would perhaps have continued to so void them had he kept the urethra of proper size, as he had previously done. But the discontinuance of the introduction of the bougie a few weeks resulted in a contraction sufficient to arrest the next calculus that attempted to pass. Upon examination, the stone was readily felt, through the integuments, near the bulb of the urethra. A sound passed through the stricture at once came in contact with the gravel. The bladder was emptied with pain and great difficulty. For two weeks or more an attempt was made to dilate the stricture, with the hope that the stone might pass. This was found impracticable, and I procured Sir Henry Thompson's divulsor, and, passing the instrument beyond the stone, opened the blades with the screw to two-thirds its dimensions; thus dilating the stricture, two calculi fell between the blades. The screw being turned off, the spring of the blades was sufficiently great to retain the gravel between them, when the instrument was removed. The instrument was again introduced, and a third calculus removed in the same way. The patient was again impressed with the importance of keeping the urethra sufficiently dilated for the passage of the small gravel that were occasionally passing.

This concludes the detail of all of the prominent features of the thirty-seven cases of urinary calculus that have passed under my observation. It will be observed that, in the thirty-four cases in which the stone was removed from the bladder, thirty-three were removed by lateral lithotomy, and in all but one the section was made with the lithotome. It may be asked if I believe that lateral lithotomy is the very best method of performing this operation? I would answer that, for me, it is; that a surgeon, whatever may be his skill, will always operate best with instruments that he is accustomed to use, and by the method with which he is most familiar. Again, I would state that statistics have demonstrated the fact that surgeons who have adopted the lateral method, whether with gorget, lithotome, or bistoury, can present as brilliant a series of lithotomies as those who have adopted any other method. If I take my results, and count out the two cases in which I operated under protest, as it were, I would only have one death in thirty-two cases—a success certainly sufficiently satisfactory.

Much could be said for and against any one method, in particular cases; but, as the object of this paper is not to discuss the advantages or the objectionable features of the several methods, I will close with the suggestion that too much has already been said by parties favoring one or the other method of operating.

